# The effect of spinal manipulation on deep experimental muscle pain in healthy volunteers

**DOI:** 10.1186/s12998-015-0069-4

**Published:** 2015-09-07

**Authors:** Søren O’Neill, Øystein Ødegaard-Olsen, Beate Søvde

**Affiliations:** Spine Centre of Southern Denmark, Lillebælt Hospital, Østre Hougvej 55, Middelfart, 5500 DK Denmark; Institute of Regional Health Research, University of Southern Denmark, Campusvej 55, Odense, 5230 DK Denmark; Stathelle Healthcentre, Brugata 10, Stathelle, 3960 Norway

## Abstract

**Background:**

High-velocity low-amplitude (HVLA) spinal manipulation is commonly used in the treatment of spinal pain syndromes. The mechanisms by which HVLA-manipulation might reduce spinal pain are not well understood, but often assumed to relate to the reduction of biomechanical dysfunction. It is also possible however, that HVLA-manipulation involves a segmental or generalized inhibitory effect on nociception, irrespective of biomechanical function. In the current study it was investigated whether a local analgesic effect of HVLA-manipulation on deep muscle pain could be detected, in healthy individuals.

**Methods and materials:**

Local, para-spinal muscle pain was induced by injection of 0.5 ml sterile, hyper-tonic saline on two separate occasions 1 week apart. Immediately following the injection, treatment was administered as either a) HVLA-manipulation or b) placebo treatment, in a randomized cross-over design. Both interventions were conducted by an experienced chiropractor with minimum 6 years of clinical experience. Participants and the researcher collecting data were blinded to the treatment allocation. Pain scores following saline injection were measured by computerized visual analogue pain scale (VAS) (0-100 VAS, 1 Hz) and summarized as a) Pain duration, b) Maximum VAS, c) Time to maximum VAS and d) Summarized VAS (area under the curve). Data analysis was performed as two-way analysis of variance with treatment allocation and session number as explanatory variables.

**Results:**

Twenty-nine healthy adults (mean age 24.5 years) participated, 13 women and 16 men. Complete data was available for 28 participants. Analysis of variance revealed no statistically significant difference between active and placebo manipulation on any of the four pain measures.

**Conclusion:**

The current findings do not support the theory that HVLA-manipulation has a non-specific, reflex-mediated local or generalized analgesic effect on experimentally induced deep muscle pain. This in turn suggests, that any clinical analgesic effect of HVLA-manipulation is likely related to the amelioration of a pre-existing painful problem, such as reduction of biomechanical dysfunction.

## Background

Spinal manipulation is commonly used in an effort to alleviate musculoskeletal pain, but the exact mechanisms by which such treatments can reduce pain are not well understood. It is commonly assumed however, that when pain arises from biomechanical dysfunction, spinal manipulation may affect pain relief through the reduction of such dysfunction. Pain relief following spinal manipulation is thus assumed to indicate curative treatment of painful biomechanical dysfunction.

A number of clinical studies now indicate, that spinal manipulation may be a prudent choice for patients with musculoskeletal pain, not least spinal pain syndromes [1]. It is difficult however, to reliably identify those patients who are most likely to benefit from spinal manipulation and it is also a challenge to demonstrate the presence of biomechanical dysfunction in a valid and reliable manner [2, 3].

A pragmatic solution which has been suggested, is a trial of a few treatments to assess the potential benefit of further spinal manipulation for a given patient [4, 5]. This is perfectly sensible, assuming that pain relief with 2–4 such treatments indicates reduction of some underlying painful mechanical lesion or dysfunction. However, if spinal manipulation has non-specific, reflex-mediated pain-inhibitory effects irrespective of the underlying cause of nociception, such pain relief may in fact simply mask the symptoms of other painful disorders, which are not appropriately treated with *high-velocity, low-amplitude* (HVLA) manipulation. In other words: If spinal manipulation has substantial local or regional, non-specific analgesic effects irrespective of the cause of pain, such treatment could potentially obfuscate serious pathology.

Any such non-specific, reflex-mediated pain-inhibitory effect is likely to be neurophysiological in nature and neural mechanisms have played a central role in many of the theoretical models of spinal manipulation. By the same token, the first detailed theoretical model of a central reflex-mediated nociceptive modulatory mechanism focused specifically on the impact of mechano-sensory input on nociceptive processing (the pain-gate theory proposed by Melzack and Wall [6]). In more recent years several studies have demonstrated that mechanical stimulation such as joint manipulation/mobilization can inhibit pain: In laboratory animal models of inflammatory joint pain Sluka and Wright [7] and Skyba et al. [8] demonstrated a hypoalgesic effect with joint mobilization, and showed that this effect is mediated by serotonin and noradrenaline sensitive pathways. Extra-cellular thalamic recordings of nociceptive specific and wide-dynamic-range neurons (which are known to be involved in pain processing in states of central pain hypersensitivity) in rats has demonstrated that HVLA manipulation raises the mechanical response threshold, and furthermore that variations in the manipulation amplitude, but not duration affects the resulting hypoalgesia [9, 10]. Song et al. [11] demonstrated an effect of daily manipulations over a week (by Activator®;) on pain from nerve-root inflammation, assessed by both behavioral pain measures and intracellular recordings in rats. These findings and other like them provide objective evidence of a central nociceptive inhibitory effect of mechanical stimuli in laboratory animals, which appear to be reliant on serotonin and noradrenaline sensitive pathways, thus hinting at a descending pain control mechanism from the periaquaductal gray matter and rostro-ventral medial medulla of the brain stem (see Lewis et al. [12] for a recent review of conditioned pain modulation in chronic pain).

Obviously, the translational value of animal models in pain research can be questioned [13, 14] but human research supports the presence of hypoalgesic effects of manipulation which are not mediated by (segmental) opioid sensitive pathways [15] and the findings of hypoalgesia, sympathetic facilitation and changes in motor-control by Vicenzino et al. [16, 17] and Sterling et al. [18] support a mechanism of descending inhibitory control from the brain stem. The later study by Bishop et al. [19] which demonstrated changes in thermal summation pain both segmentally and caudal to the manipulation also support descending inhibition as a likely mechanism of HVLA induced hypoalgesia.

It seems plausible then, that HVLA manipulation could exert a diffuse hypoalgesic effect on the basis of descending inhibitory control from the brain stem. A number of factors may have influenced previous findings, including (but not limited to) the study population, the time scale within which the effect of HVLA-manipulation is sought and the method with which pain sensitivity has been examined.

Whereas the specific cause and nature of pain in clinical spinal pain states is often undetermined, experimental pain can be induced in a manner which is controlled and well understood – researchers inducing experimental pain know the precise nature and tissue site of the pain they have induced and research indicates that spinal manipulation has a significant analgesic effect on such experimental pain whether induced by pressure, mechanical stretching, capsaicin or electrical stimulation [20, 21]. Most studies however, have examined and demonstrated that the effect is evident at the same spinal level or in the neighboring region of the experimental pain stimulus and HVLA manipulation.

The literature summarized by the reviews by Millan et al. [20] and Coronado et al. [21] examines the effects of spinal manipulation on *experimental* pain. Some of the reviewed literature is on healthy volunteers [19, 22–29], but most [16–18, 30–44] of the studies actually examine populations of patients with *clinical* pain conditions ranging from mechanical neck pain and low back pain to tender-points and latent trigger points. It is therefor possible, that any observed local hypoalgesic effect of spinal manipulation in those studies, is due to an effect on the underlying painful clinical condition itself or changes in central pain modulation specifically related to those clinical conditions.

In order to gain a better understanding of the mechanisms by which spinal manipulation may afford such hypoalgesic effects, it is necessary to distinguish between effects inherent to the manipulation itself, and effects mediated by the potential influence on an underlying clinical pain condition – is any observed difference in pain sensitivity caused by a diffuse anti-nociceptive effect of manipulation, or by reduction of some underlying (mechanical) painful condition, such as latent trigger points?

Furthermore, the manner in which experimental pain sensitivity is tested may prove important. Research suggests that different aspects of the pain experience are not simple correlates [45–50]; deep and superficial pain sensitivity may vary, as may sensitivity to different pain modalities (thermal, chemical, mechanical, etc) and the choice of pain indicators (thresholds, distribution, duration, modulation, quality, etc) may also be important.

The purpose of this study was examine the immediate, local, deep-tissue hypoalgesic effect of spinal manipulation in healthy volunteers with no assumption of underlying painful clinical or sub-clinical conditions.

## Method and materials

### Design

A randomized, controlled, crossover study.

### Recruitment

Participants were recruited from the student population of the chiropractic degree program at the University of Southern Denmark. Only healthy volunteers without known contraindications to spinal manipulation were invited to participate.

As part of the university degree program, all chiropractic students are evaluated by a certified chiropractor during their 3rd semester, in order to detect possible contradictions for HVLA manipulations. Four study participants were recruited from the 2nd semester and prior to study participation these were interviewed and examined for contraindications for HVLA manipulation by a senior chiropractor at The Spinecentre of Southern Denmark, Lillebaelt Hospital, Denmark.

Participants with chronic pain, previous back surgery, current back problems, somatic or psychological conditions were excluded.

Study participants were recruited through verbal information in the classroom and written information was sent electronically to those who expressed an interest in participating.

The Regional Scientific Ethical Committee for Southern Denmark approved the study (S-0080137) and written consent was obtained from all participants.

### Experimental procedure

The experiment was carried out at the Spinecentre of Southern Denmark, Lillebaelt Hospital. Two assistants (graduate students) and two senior chiropractors from the department conducted the study.

Participants were informed that they would receive two effective, but different treatments and that the aim of the study was to compare any difference in effectiveness between them. All participants were treated on two separate occasions at least 1 week apart and treatment sessions were identical apart from the actual manual treatment performed: a) active HVLA-manipulation treatment, and b) placebo treatment with an Activator instrument. Participants were not informed that one of the treatments was a placebo.

Treatments at both the first and second session were directed at the 6th thoracic level. Allocation of treatment was done randomly according to a pre-hoc computer generated list in which treatment and session was randomized, but balanced.

On each occasion, in preparation of the test procedure, assistant 1 (BS) identified the spinous process of the 6th thoracic segment through palpation (counting down from the bony landmark of the C7 spinous process). The T6 segment was marked with a pen and anesthetic cream (EMLA cream, Astra Zeneca – an eutectic mixture of the local anesthetics Lidocain and Prilocain) was applied para-spinally on the right side of the T6 spinous process and covered by a patch with self-adhesive borders (Tegaderm, 5×7 cm) to keep the cream in place. The anesthetic cream was applied to ensure that experimental pain was induced primarily from deep tissues and not from needle-puncture of superficial structures.

After 20 min, assistant 2 (ØØO) removed the patch, wiped off the anesthetic cream and instructed the participant in the use of the computerized visual analogue pain scale, before asking the participant to lie prone on a chiropractic treatment table.

The injection point was the same for all participants; para-spinally at T6 (center of the anesthetized area) which was wiped down twice with alcohol swaps, as per standard aseptic injection technique. Assistant 2 proceeded to aspirate and gradually (over approx 2 s) inject 0.5 ml sterile hyper-tonic saline solution (Sodium SAD 1 mmol/ml) at room temperature, into the right T6 para-spinal musculature at approximately 2 cm depth. Immediately thereafter, assistant 2 left the room and one of the two senior chiropractors delivered the allocated treatment.

Placebo treatment was performed immediately next to the injection site. The chiropractor stabilized the chosen segment with the left hand, while holding the activator in the right hand, placing it on the anesthetized skin on the right side of the transverse process. The participant was asked to take a deep breath. Upon exhalation the Activator was pressed twice, releasing two ‘click’ sounds, while giving no mechanical impulse (amplitude = 0 mm).

HVLA-manipulation was performed with bilateral thenar contact (Carver bridge) on both transverse processes on the T6 segment. The participant was asked to take a deep breath and upon exhalation the HVLA manipulation was administered over the segment resulting in a mechanical impulse, in most instances with an accompanying articular cavitation.

**Fig. 1:**
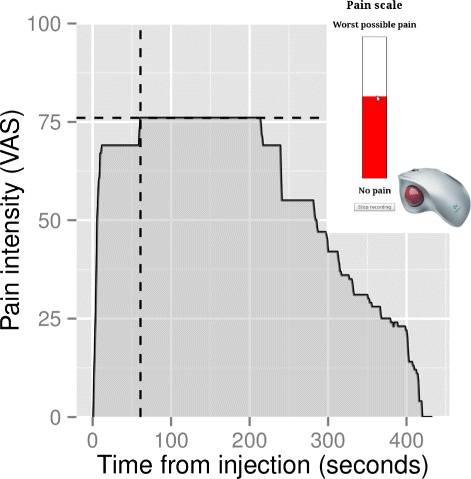
Illustration of computerized VAS data. Pain intensity was measured using an on-screen visual analogue pain scale (insert in figure) controlled by the participant with a common computer trackball. The pain intensity was sampled with a frequency of 1 Hz and the resulting time-series data is illustred in the graph

Immediately after administering the treatment, the chiropractor left the room and (the blinded) assistant 2 returned. Assistant 2 ensured, that the participant indicated the intensity of pain from the saline injection as it developed over time (computerized VAS). The computerized VAS consisted of a scale marked ‘No pain’ at one end and ‘Worst possible pain’ at the other, corresponding to a value between 0 and 100 respectively. The VAS was sampled with a frequency of 1 Hz and data was stored electronically, allowing for a time-series of VAS measurements (see Fig. 1 for an illustration). During the recording, there was complete silence and no activity in the treatment room.

Data recording was considered complete when the patient indicated that the pain declined to near 0 – typically within 3–5 min.

The participants were all chiropractic students with personal knowledge of spinal manipulative procedures, which challenges the validity of the chosen placebo treatment. For that reason, upon completion of the experimental part of the study, 14 participants were randomly selected for a phone interview and questioned if they had, at any point been aware that one of the treatments was a placebo treatment.

## Data

The VAS data was summarized as four outcome variables: a) duration of pain, b) max VAS value, c) time from pain onset to max VAS value and d) accumulated area under the curve (VAS over time). See Fig. 1 for an illustration.

## Statistical analysis

Data are presented as median values with minimum, maximum and 1 ^*st*^ and 3 ^*rd*^ quartiles. The results are analyzed with two-way Anova with treatment allocation and session number as explanatory variables. Homogeneity of variance was analyzed with Fligner-Killeen test. An alpha level of 5 % was considered statistically significant. Analysis was performed using R version 3.0.2 (2013-09-25) for Linux [51].

## Results

A total of 29 participants took part in the study; 13 women and 16 men (mean age 24.5 years).

No complications or side effects were reported by any of the participants and there were no dropouts. Due to technical computer problems however, raw data for 1 participant (male) was lost, leaving complete data for both sessions for 28 subjects.

Fourteen (14) participants received the active treatment on the first session (and placebo on the second), and vice versa.

The median duration of saline induced pain was 219 s (min = 101, 1. quartile = 178, 3. quartile = 274, max = 433), the median maximum VAS was 48 VAS (min = 7, 1.quartile = 35, 3.quartile = 66, max = 98), the median time from pain onset to max VAS was 44.5 s (min = 1, 1.quartile = 27, 3.quartile = 90.25, max = 143) and the median area-under-the-curve was 6117 VAS ×seconds (min = 1019, 1.quartile = 3572, 3.quartile = 9182, max = 25010).

Fligner-Killeen test of homogeneity of variance for each of the four outcome variables, by each of the two explanatory variables yielded P values between 0.26 and 0.92, i.e. no heteroscedasticity of variance was observed.

Two-way analysis of variance revealed no significant differences in the 4 outcome variables by either explanatory variable, with one exception: A significant effect (*p* = 0.005) of session number on duration of pain was found, with longer duration of pain on the first session (mean = 256 s, 95 % QI [227;285]) compared to the second session (mean = 202 s, 95 % QI [178;226]).

A post-hoc power-calculation (based on paired t-test) revealed, that with *n* = 28, alpha = 0.05 and beta = 0.8, a moderate effect size (0.55) would have been detected if present.

None of the 14 randomly selected participants interviewed following the experiment indicated that they had any suspicion that the Activator treatment had been a placebo treatment.

## Discussion

If the effects of HVLA-manipulation was due primarily to an immediate, non-specific and reflex-mediated inhibition of nociception, we would have expected to observe a difference in saline induced pain in the current study between active and placebo treatment, but no such difference was observed.

***Current findings in relation to previous publications*** At first, the present findings appear to be in contrast to most of the published literature on HVLA-manipulation and experimentally induced pain, as summarized in the two recent reviews by Millan et al. and Coronado et al. [20, 21]. The authors reported significant pain inhibitory effects of HVLA-manipulation on experimentally induced pain.

However, the published literature was mostly based on populations with clinical or sub-clinical conditions [16–18, 30–44], i.e. patients with clinical pain states such as neck and low back pain, or non-clinical populations in which HVLA-manipulation was directed at tissues identified as pain hypersensitive (tender-points, trigger points). In those instances, it is unclear whether any effect of HVLA-manipulation should be ascribed to a non-specific reflex-mediated pain-inhibitory effect of the manual procedure itself, or a specific effect on the (sub-)clinical condition. In the present study, manipulation was not directed at any such clinical or sub-clinical condition and all participants were tested (arbitrarily) at the T6 segment. Any effect of HVLA-manipulation, had it been observed in the present study could thus have been ascribed to such non-specific, reflex-mediated pain inhibition.

Of the previous studies investigating healthy, pain free participants [19, 22–29] reviewed by Millan et al. and Coronado et al., the majority relied on pressure-pain thresholds and reported contradictory findings: Fernández-de-las-Penãs et al. [22, 23] used placebo-controlled designs and demonstrated an increase in pressure pain thresholds following cervical manipulation. This is in contrast to the controlled studies by Hamilton [25] and Soon [27], who reported no effect of manipulation on pressure pain thresholds. The study by Bishop et al. [19] which also involved a control group found a difference in thermal sensory summation between groups, but no difference in pressure pain or thermal pain thresholds.

The studies by Krouwel et al. [26] and Willet et al. [29] are harder to interpret as they did not involve clear-cut control groups: There is a tendency for experimental pain sensitivity to be higher on the first test, compared to subsequent tests which was also the finding in several of the papers discussed here – this underlines the importance of a control group in paired *before/after* pain sensitivity studies. In any case, the studies by Krouwel et al. [26] and Willet et al. [29] are similar in several respects, and while they both include a treatment group receiving simple sustained (*quasi-static*) pressure which could be interpreted as a minimalist intervention control group, this is not clearly stated. In any case, no group differences in pain thresholds are reported in either study.

Finally, the studies by George et al. [24] and Terrett and Vernon [28] both employed superficial pain stimuli (thermal and electrical, respectively). Whereas Terrett and Vernon reported a 140 % increase in superficial pain tolerance thresholds following manipulation, George et al. reported only few differences between those receiving manipulation, performing extension exercises or simply riding a stationary bicycle. Clearly, the literature on manipulation induced hypoalgesia in *healthy, pain-free subjects* is not concordant.

Care should be exercised when comparing animal and human pain research, but as described in the *Background* section, animal research indicates the presence of a non-specific anti-nociceptive effect of manipulation, possibly based on descending inhibitory control. The current findings did not demonstrate such an effect.

***Differences between the present methodology and previous publications*** The present study differs from the previously published literature on HVLA manipulation in the choice of pain stimulus: intra-muscular injection of hyper-tonic saline. Most of the previously published studies have used either pressure pain thresholds or superficial (skin) stimuli [20, 21]. Injection of hyper-tonic saline is a common experimental model of muscle pain [52], albeit less common than the ubiquitous pressure algometer. Slater et al. [53] also induced experimental pain by injection of hyper-tonic saline, albeit in adjunct to *delayed onset muscle soreness (DOMS)* of the extensor carpi radialis muscle and reported no difference in saline pain intensity, nor in pressure pain sensitivity following *mobilization-with-movement* manual therapy compared to placebo. In this respect, the present study is in alignment with the methodology and findings of Slater et al.

Although the specific seat and nature of most clinical spinal pain states for which spinal manipulation is used is unknown, it is rarely thought to originate in superficial structures, such as skin, subcutaneous connective tissues, etc. Furthermore, spinal manipulation is typically administered in an attempt to affect deep tissues such as facet joints, intervertebral disks and segmental musculature, but obviously also affects superficial tissues. The use of intra-muscular injection of hyper-tonic saline and a superficial anesthetic cream in the present study was chosen to ensure that the pain stimulus was indeed primarily delivered to deep spinal tissues.

The anesthetic cream was applied to reduced the superficial pain of the needle puncture and thus ensure that the induced experimental pain stemmed primarily from the deep intra-muscular saline. Whereas the cream will not have affected the deep experimental pain to any appreciable degree, it could arguably have affected the *profile* of sensory input in relation to the manipulation. The current study was not designed to cast light on any (potential) effect of superficial sensation or anesthesia on the effects of spinal manipulation, but this might be worth investigating in future research.

Conversely, the clinical pain for which HVLA spinal manual therapy is delivered, is of longer duration than the experimental pain induced in the current study – typically 2–4 min. This is a potential limitation of the current design and if, as some animal research indicates HVLA manipulation exerts anti-nociceptive effects through descending inhibitory control, a much longer time-frame is likely necessary to demonstrate such effects.

As there is no consensus on which experimental pain stimulus best simulate clinical pain or which tests most reliably reveals changes in pain sensitivity, a selection of diverse pain stimuli and measures are often recommended. In the present study only a single pain stimuli was used, however it could be argued that deep muscle pain more closely resembles clinical back pain than mechanical pressure or superficial thermal or electrical stimuli. The outcome measures used in the present study were related to several aspects of the pain experience: pain onset, intensity and duration. Other aspects such as pain detection- and tolerance threshold, spatial distribution, qualitative pain description, conditioned pain modulation and others could equally have been relevant, but as always a balance had to be struck between feasibility and methodological rigor.

Several studies in the pain literature conclude that pain sensitivity is complex and multi-modal, and that adequate assessment requires a battery of different quantitative sensory tests of pain sensitivity [45–50, 54]. The only study published on the *generalizability* of quantitative sensory testing of experimental pain [54], suggests that composite pain scores, such as those affected by e.g. both pain intensity and duration, offer greater generalizability than single scores such as e.g. pain threshold. We can not exclude however, that group differences could have been revealed by other quantitative sensory pain tests.

As an incidental finding, significantly longer pain duration on the first session, compared to the second session, was observed. This is not unusual in experimental pain research and is likely due to a degree of anxiety and unfamiliarity with the pain stimulus in the first session. For this reason some researchers employ an ‘introductory’-session to familiarize study participants with the stimulus, some time well in advance of the actual study. This was not the case in the present study; instead the effect was countered by random allocation of treatments to session 1 and 2.

***Study population*** A potential limitation of the current study was the study population: students recruited from the chiropractic program at the University of Southern Denmark. This represents a potential bias, as the study population has prior knowledge of manipulative techniques and therefor might be in a position to recognize the placebo treatment as such. The study population was chosen for practicality, availability and economy, despite the potential sources of bias. According to Vernon et al. [55] it is difficult, but possible to construct a valid placebo treatment for manual procedures if it meets the following criteria; 1) the intervention should have no significant treatment effect and 2) the subject should be unable to determine whether they have received a sham treatment. All participants in the current study were unaware of the order of treatment and the existence of a placebo intervention, and none of the 14 participants, randomly chosen for interview after the study were aware of the use of a placebo treatment. We would thus argue that the inactivated Activator treatment represents a true placebo treatment in the current study.

***Interpretation of findings***

The current findings should not be over-interpreted. They indicate however, that HVLA-manipulation does not affect an immediate non-specific, reflex-mediated inhibition of deep-tissue segmental pain. Indirectly the findings thus lend support to the assumption that pain relief from HVLA-manipulation is likely to represent reduction of pain from some underlying painful condition, e.g. biomechanical dysfunction. This, in turn, suggests that the approach recommended by Leboeuf-Yde et al. and Axén et al. [4, 5], with a trial of 2–4 treatments to assess the effect of HVLA-manipulation is indeed a pragmatic and safe one. As stated above however, necessary choices regarding pain stimulus, pain measures, time frame etc should temper the conclusions based on a single study.

Future studies should take into consideration both the study population, the test- and treatment-sites and the chosen outcome measures. When quantifying experimentally induced pain in participants with painful clinical conditions, it becomes difficult to separate the intrinsic effects of HVLA manipulation from those of a clinical effect on the underlying cause of pain. Furthermore, if the intent is to disentangle the underlying neurological mechanisms of HVLA-manipulation induced hypoalgesia, test sites should be chosen which are appropriate for the hypothesis; local, segmental or generalized effects. And finally, the choice of pain measures is important and a battery of tests covering both different pain modalities and response domains is to be preferred.

## Conclusion

The current study indicates, that HVLA-manipulation does not have an immediate non-specific, reflex-mediated local hypoalgesic effect on experimentally induced deep para-spinal muscle pain in healthy volunteers after skin anesthesia.
